# *N*-(2-Bromo­phen­yl)-2-(naphthalen-1-yl)acetamide

**DOI:** 10.1107/S1600536812034423

**Published:** 2012-08-08

**Authors:** Hoong-Kun Fun, Ching Kheng Quah, Prakash S. Nayak, B. Narayana, B. K. Sarojini

**Affiliations:** a X-ray Crystallography Unit, School of Physics, Universiti Sains Malaysia, 11800 USM, Penang, Malaysia; b Department of Studies in Chemistry, Mangalore University, Mangalagangotri 574 199, India; c Department of Chemistry, P.A. College of Engineering, Nadupadavu, Mangalore 574 153, India

## Abstract

In the title compound, C_18_H_14_BrNO, the naphthalene ring system [maximum deviation = 0.015 (3) Å] forms a dihedral angle of 67.70 (10)° with the benzene ring. In the crystal, mol­ecules are linked by N—H⋯O hydrogen bonds into *C*(4) chains propagating in [100]. A C—H⋯O inter­action reinforces the chain connectivity, generating an *R*_2_^1^(6) loop.

## Related literature

For general background to and related structures of the title compound, see: Fun *et al.* (2010 ▶, 2011*a* ▶,*b* ▶, 2012 ▶). For the stability of the temperature controller used for the data collection, see: Cosier & Glazer (1986 ▶). For hydrogen-bond motifs, see: Bernstein *et al.* (1995 ▶).
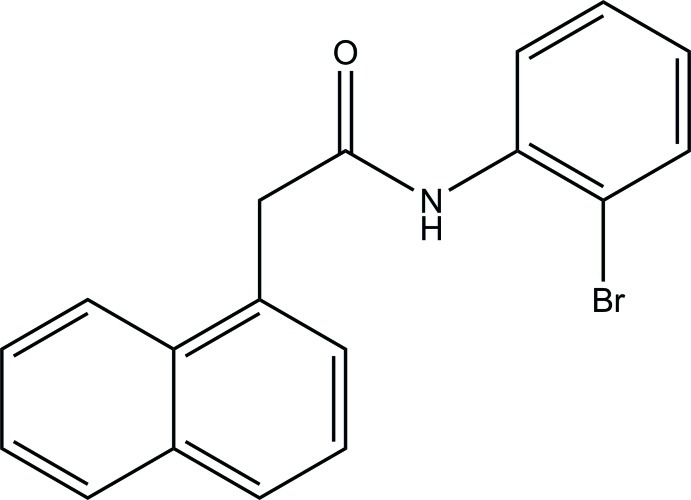


## Experimental

### 

#### Crystal data

C_18_H_14_BrNO
*M* *_r_* = 340.21
Orthorhombic, 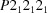

*a* = 4.7603 (1) Å
*b* = 11.4614 (3) Å
*c* = 26.6255 (6) Å
*V* = 1452.68 (6) Å^3^
*Z* = 4
Mo *K*α radiation
μ = 2.83 mm^−1^
*T* = 100 K
0.32 × 0.16 × 0.13 mm

#### Data collection

Bruker SMART APEXII CCD diffractometer
Absorption correction: multi-scan (*SADABS*; Bruker, 2009 ▶) *T*_min_ = 0.463, *T*_max_ = 0.719
16642 measured reflections
3864 independent reflections
3516 reflections with *I* > 2σ(*I*)
*R*_int_ = 0.031

#### Refinement

*R*[*F* ^2^ > 2σ(*F* ^2^)] = 0.029
*wR*(*F* ^2^) = 0.061
*S* = 1.03
3864 reflections
195 parameters
H atoms treated by a mixture of independent and constrained refinement
Δρ_max_ = 0.63 e Å^−3^
Δρ_min_ = −0.33 e Å^−3^
Absolute structure: Flack (1983 ▶), 1587 Friedel pairs
Flack parameter: 0.001 (8)

### 

Data collection: *APEX2* (Bruker, 2009 ▶); cell refinement: *SAINT* (Bruker, 2009 ▶); data reduction: *SAINT*; program(s) used to solve structure: *SHELXTL* (Sheldrick, 2008 ▶); program(s) used to refine structure: *SHELXTL*; molecular graphics: *SHELXTL*; software used to prepare material for publication: *SHELXTL* and *PLATON* (Spek, 2009 ▶).

## Supplementary Material

Crystal structure: contains datablock(s) global, I. DOI: 10.1107/S1600536812034423/hb6923sup1.cif

Structure factors: contains datablock(s) I. DOI: 10.1107/S1600536812034423/hb6923Isup2.hkl

Supplementary material file. DOI: 10.1107/S1600536812034423/hb6923Isup3.cml

Additional supplementary materials:  crystallographic information; 3D view; checkCIF report

## Figures and Tables

**Table 1:** Hydrogen-bond geometry (Å, °)

*D*—H⋯*A*	*D*—H	H⋯*A*	*D*⋯*A*	*D*—H⋯*A*
N1—H1*N*1⋯O1^i^	0.86 (3)	2.03 (3)	2.855 (3)	163 (2)
C11—H11*A*⋯O1^i^	0.99	2.55	3.279 (3)	130

Symmetry code: (i) 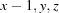.

**Fig. 1.**
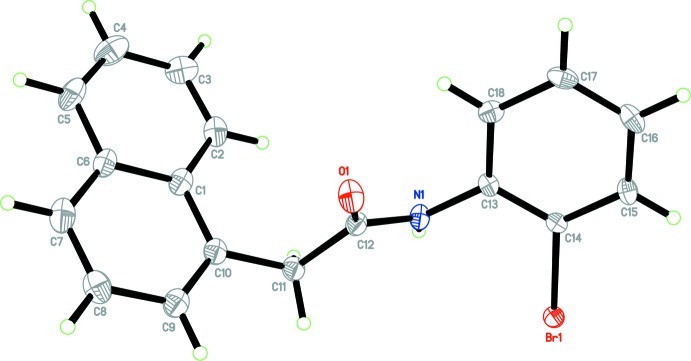
The molecular structure of the title compound showing 40% probability displacement ellipsoids for non-H atoms.

**Fig. 2.**
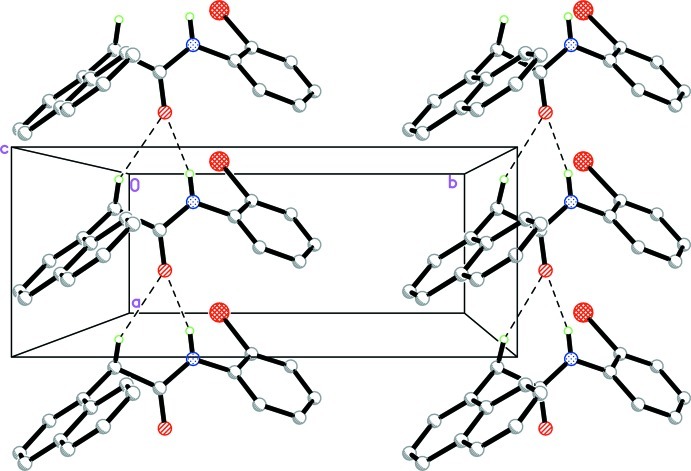
The crystal structure of the title compound, viewed along the c axis. H atoms not involved in hydrogen bonds (dashed lines) have been omitted for clarity.

## supplementary crystallographic information

### Comment

In continuation of our work on synthesis of amides (Fun *et al.*, 2010, 2011*a*, 2011*b*, 2012), we report herein the crystal structure of the title compound.

The molecular structure is shown in Fig. 1. Bond lengths are comparable to related structures (Fun *et al.*, 2010, 2011*a*, 2011*b*, 2012). The naphthalene ring system (C1-C10, maximum deviation of 0.015 (3) Å at atom C9) forms a dihedral angle of 67.70 (10)° with the benzene ring (C13-C18).

In the crystal structure, Fig. 2, molecules are linked *via* N1–H1N1···O1 and C11–H11A···O1 hydrogen bonds (Table 1) into one-dimensional [100] chains which contain R_2_^1^ (6) ring motifs (Bernstein *et al.*, 1995).

### Experimental

1-Naphthaleneacetic acid (0.186 g, 1 mmol), 2-bromoaniline (0.1 ml, 1 mmol) and 1-ethyl-3-(3-dimethylaminopropyl)-carbodiimide hydrochloride (1.0 g, 0.01 mol) were dissolved in dichloromethane (20 ml). The mixture was stirred in presence of triethylamine at 273 K for about 3 h. The contents were poured into 100 ml of ice-cold aqueous hydrochloric acid with stirring. The concotion was then extracted thrice with dichloromethane. The organic layer was washed with saturated NaHCO_3_ solution and brine solution, dried and concentrated under reduced pressure to give the title compound. Colourless blocks were grown from toluene solution by the slow evaporation method (*m.p.*: 421K).

### Refinement

Atom H1N1 was located in a difference Fourier map and refined freely [N–H = 0.86 (3) Å]. The remaining H atoms were positioned geometrically and refined using a riding model with C–H = 0.95 or 0.99 Å and *U*_iso_(H) = 1.2 *U*_eq_(C). The reported Flack parameter was obtained by TWIN/BASF procedure in *SHELXL* (Sheldrick, 2008).

### Crystal data

**Table:**

C_18_H_14_BrNO	*F*(000) = 688
*M**_r_* = 340.21	*D*_x_ = 1.556 Mg m^−^^3^
Orthorhombic, *P*2_1_2_1_2_1_	Mo *K*α radiation, λ = 0.71073 Å
Hall symbol: P 2ac 2ab	Cell parameters from 8019 reflections
*a* = 4.7603 (1) Å	θ = 2.3–30.8°
*b* = 11.4614 (3) Å	µ = 2.83 mm^−^^1^
*c* = 26.6255 (6) Å	*T* = 100 K
*V* = 1452.68 (6) Å^3^	Block, colourless
*Z* = 4	0.32 × 0.16 × 0.13 mm

### Data collection

**Table:**

Bruker SMART APEXII CCD diffractometer	3864 independent reflections
Radiation source: fine-focus sealed tube	3516 reflections with *I* > 2σ(*I*)
Graphite monochromator	*R*_int_ = 0.031
φ and ω scans	θ_max_ = 29.0°, θ_min_ = 1.5°
Absorption correction: multi-scan (*SADABS*; Bruker, 2009)	*h* = −6→6
*T*_min_ = 0.463, *T*_max_ = 0.719	*k* = −15→15
16642 measured reflections	*l* = −36→36

### Refinement

**Table:**

Refinement on *F*^2^	Secondary atom site location: difference Fourier map
Least-squares matrix: full	Hydrogen site location: inferred from neighbouring sites
*R*[*F*^2^ > 2σ(*F*^2^)] = 0.029	H atoms treated by a mixture of independent and constrained refinement
*wR*(*F*^2^) = 0.061	*w* = 1/[σ^2^(*F*_o_^2^) + (0.0272*P*)^2^ + 0.2223*P*] where *P* = (*F*_o_^2^ + 2*F*_c_^2^)/3
*S* = 1.03	(Δ/σ)_max_ = 0.001
3864 reflections	Δρ_max_ = 0.63 e Å^−^^3^
195 parameters	Δρ_min_ = −0.33 e Å^−^^3^
0 restraints	Absolute structure: Flack (1983), 1587 Friedel pairs
Primary atom site location: structure-invariant direct methods	Flack parameter: 0.001 (8)

### Special details

**Table:**

Experimental. The crystal was placed in the cold stream of an Oxford Cryosystems Cobra open-flow nitrogen cryostat (Cosier & Glazer, 1986) operating at 100.0 (1) K.
Geometry. All esds (except the esd in the dihedral angle between two l.s. planes) are estimated using the full covariance matrix. The cell esds are taken into account individually in the estimation of esds in distances, angles and torsion angles; correlations between esds in cell parameters are only used when they are defined by crystal symmetry. An approximate (isotropic) treatment of cell esds is used for estimating esds involving l.s. planes.
Refinement. Refinement of F^2^ against ALL reflections. The weighted R-factor wR and goodness of fit S are based on F^2^, conventional R-factors R are based on F, with F set to zero for negative F^2^. The threshold expression of F^2^ > 2sigma(F^2^) is used only for calculating R-factors(gt) etc. and is not relevant to the choice of reflections for refinement. R-factors based on F^2^ are statistically about twice as large as those based on F, and R- factors based on ALL data will be even larger.

### Fractional atomic coordinates and isotropic or equivalent isotropic displacement parameters (Å^2^)

**Table:**

	*x*	*y*	*z*	*U*_iso_*/*U*_eq_	
Br1	0.44680 (5)	0.197949 (18)	0.766005 (8)	0.01881 (6)	
O1	1.1551 (4)	0.32404 (15)	0.64580 (7)	0.0288 (4)	
N1	0.7212 (5)	0.25292 (18)	0.66352 (8)	0.0205 (4)	
C1	0.9798 (6)	0.4924 (2)	0.54712 (8)	0.0243 (5)	
C2	0.8336 (6)	0.3968 (2)	0.52540 (10)	0.0290 (6)	
H2A	0.7122	0.3512	0.5458	0.035*	
C3	0.8643 (7)	0.3692 (3)	0.47576 (10)	0.0362 (7)	
H3A	0.7616	0.3060	0.4618	0.043*	
C4	1.0465 (8)	0.4340 (3)	0.44572 (10)	0.0404 (7)	
H4A	1.0702	0.4133	0.4114	0.048*	
C5	1.1896 (7)	0.5257 (3)	0.46455 (10)	0.0342 (7)	
H5A	1.3099	0.5691	0.4431	0.041*	
C6	1.1646 (6)	0.5586 (2)	0.51556 (9)	0.0254 (5)	
C7	1.3132 (7)	0.6544 (2)	0.53578 (11)	0.0327 (6)	
H7A	1.4370	0.6981	0.5151	0.039*	
C8	1.2791 (6)	0.6844 (2)	0.58521 (10)	0.0318 (6)	
H8A	1.3788	0.7488	0.5989	0.038*	
C9	1.0935 (6)	0.6184 (2)	0.61591 (9)	0.0274 (6)	
H9A	1.0688	0.6408	0.6500	0.033*	
C10	0.9508 (6)	0.52492 (19)	0.59833 (8)	0.0237 (5)	
C11	0.7656 (6)	0.45304 (19)	0.63269 (9)	0.0238 (5)	
H11A	0.5846	0.4374	0.6156	0.029*	
H11B	0.7254	0.4984	0.6635	0.029*	
C12	0.9006 (6)	0.3379 (2)	0.64713 (8)	0.0211 (6)	
C13	0.8129 (5)	0.14552 (19)	0.68404 (8)	0.0169 (5)	
C14	0.7098 (5)	0.10589 (17)	0.73006 (8)	0.0159 (4)	
C15	0.7974 (5)	0.00074 (19)	0.75029 (8)	0.0202 (5)	
H15A	0.7212	−0.0260	0.7812	0.024*	
C16	0.9963 (5)	−0.06519 (18)	0.72528 (8)	0.0244 (6)	
H16A	1.0604	−0.1365	0.7394	0.029*	
C17	1.1024 (5)	−0.0273 (2)	0.67953 (9)	0.0240 (6)	
H17A	1.2386	−0.0729	0.6623	0.029*	
C18	1.0100 (5)	0.07691 (19)	0.65886 (8)	0.0207 (5)	
H18A	1.0815	0.1018	0.6273	0.025*	
H1N1	0.543 (6)	0.261 (2)	0.6612 (8)	0.015 (6)*	

### Atomic displacement parameters (Å^2^)

**Table:**

	*U*^11^	*U*^22^	*U*^33^	*U*^12^	*U*^13^	*U*^23^
Br1	0.01624 (11)	0.01879 (9)	0.02138 (9)	0.00023 (10)	0.00331 (10)	0.00085 (8)
O1	0.0139 (9)	0.0299 (10)	0.0427 (10)	0.0035 (8)	0.0034 (9)	0.0116 (8)
N1	0.0126 (11)	0.0246 (10)	0.0244 (9)	0.0045 (9)	0.0025 (10)	0.0081 (8)
C1	0.0171 (14)	0.0310 (11)	0.0249 (10)	0.0080 (11)	−0.0005 (11)	0.0072 (9)
C2	0.0178 (13)	0.0312 (13)	0.0380 (14)	0.0009 (12)	−0.0006 (13)	0.0094 (11)
C3	0.0269 (17)	0.0460 (16)	0.0359 (14)	0.0036 (14)	−0.0049 (13)	−0.0062 (12)
C4	0.0273 (15)	0.0627 (19)	0.0312 (12)	0.0067 (18)	0.0001 (15)	−0.0022 (12)
C5	0.0229 (15)	0.0514 (17)	0.0282 (13)	0.0013 (14)	0.0047 (13)	0.0095 (12)
C6	0.0188 (13)	0.0313 (13)	0.0261 (11)	0.0052 (12)	0.0023 (12)	0.0084 (10)
C7	0.0226 (15)	0.0352 (13)	0.0404 (14)	0.0043 (13)	0.0041 (13)	0.0134 (11)
C8	0.0249 (15)	0.0285 (14)	0.0419 (14)	−0.0021 (13)	−0.0034 (12)	0.0033 (11)
C9	0.0242 (17)	0.0307 (12)	0.0271 (11)	0.0073 (12)	0.0011 (12)	0.0060 (9)
C10	0.0189 (12)	0.0245 (11)	0.0278 (10)	0.0071 (12)	0.0013 (13)	0.0058 (8)
C11	0.0214 (15)	0.0234 (11)	0.0264 (11)	0.0057 (11)	0.0021 (11)	0.0045 (9)
C12	0.0202 (16)	0.0237 (11)	0.0195 (10)	0.0030 (10)	0.0020 (11)	0.0028 (8)
C13	0.0116 (12)	0.0168 (10)	0.0222 (10)	−0.0002 (9)	−0.0031 (10)	0.0016 (8)
C14	0.0123 (11)	0.0158 (9)	0.0197 (9)	−0.0006 (8)	−0.0020 (11)	−0.0013 (8)
C15	0.0157 (12)	0.0187 (10)	0.0262 (10)	−0.0041 (10)	−0.0027 (10)	0.0052 (8)
C16	0.0170 (15)	0.0169 (10)	0.0393 (13)	−0.0006 (9)	−0.0036 (12)	0.0034 (8)
C17	0.0140 (15)	0.0231 (11)	0.0349 (12)	0.0026 (10)	−0.0006 (11)	−0.0088 (9)
C18	0.0146 (15)	0.0267 (11)	0.0210 (9)	0.0031 (10)	−0.0019 (10)	−0.0022 (8)

### Geometric parameters (Å, º)

**Table:**

Br1—C14	1.896 (2)	C8—C9	1.422 (4)
O1—C12	1.223 (3)	C8—H8A	0.9500
N1—C12	1.367 (3)	C9—C10	1.352 (4)
N1—C13	1.416 (3)	C9—H9A	0.9500
N1—H1N1	0.86 (3)	C10—C11	1.514 (3)
C1—C10	1.420 (3)	C11—C12	1.517 (3)
C1—C2	1.421 (4)	C11—H11A	0.9900
C1—C6	1.434 (3)	C11—H11B	0.9900
C2—C3	1.367 (4)	C13—C18	1.396 (3)
C2—H2A	0.9500	C13—C14	1.396 (3)
C3—C4	1.394 (4)	C14—C15	1.384 (3)
C3—H3A	0.9500	C15—C16	1.382 (3)
C4—C5	1.349 (4)	C15—H15A	0.9500
C4—H4A	0.9500	C16—C17	1.388 (3)
C5—C6	1.415 (4)	C16—H16A	0.9500
C5—H5A	0.9500	C17—C18	1.386 (3)
C6—C7	1.412 (4)	C17—H17A	0.9500
C7—C8	1.370 (4)	C18—H18A	0.9500
C7—H7A	0.9500		
			
C12—N1—C13	123.4 (2)	C9—C10—C1	119.4 (2)
C12—N1—H1N1	121.0 (16)	C9—C10—C11	121.0 (2)
C13—N1—H1N1	115.6 (16)	C1—C10—C11	119.6 (2)
C10—C1—C2	123.1 (2)	C10—C11—C12	112.3 (2)
C10—C1—C6	118.9 (2)	C10—C11—H11A	109.1
C2—C1—C6	118.0 (2)	C12—C11—H11A	109.1
C3—C2—C1	121.3 (3)	C10—C11—H11B	109.1
C3—C2—H2A	119.3	C12—C11—H11B	109.1
C1—C2—H2A	119.3	H11A—C11—H11B	107.9
C2—C3—C4	119.9 (3)	O1—C12—N1	122.4 (2)
C2—C3—H3A	120.1	O1—C12—C11	121.7 (2)
C4—C3—H3A	120.1	N1—C12—C11	115.8 (2)
C5—C4—C3	121.0 (3)	C18—C13—C14	118.3 (2)
C5—C4—H4A	119.5	C18—C13—N1	120.8 (2)
C3—C4—H4A	119.5	C14—C13—N1	120.9 (2)
C4—C5—C6	121.5 (3)	C15—C14—C13	121.2 (2)
C4—C5—H5A	119.3	C15—C14—Br1	119.14 (17)
C6—C5—H5A	119.3	C13—C14—Br1	119.61 (16)
C7—C6—C5	122.1 (2)	C16—C15—C14	119.7 (2)
C7—C6—C1	119.7 (2)	C16—C15—H15A	120.2
C5—C6—C1	118.2 (2)	C14—C15—H15A	120.2
C8—C7—C6	120.1 (3)	C15—C16—C17	120.1 (2)
C8—C7—H7A	119.9	C15—C16—H16A	120.0
C6—C7—H7A	119.9	C17—C16—H16A	120.0
C7—C8—C9	119.5 (3)	C18—C17—C16	120.2 (2)
C7—C8—H8A	120.2	C18—C17—H17A	119.9
C9—C8—H8A	120.2	C16—C17—H17A	119.9
C10—C9—C8	122.3 (2)	C17—C18—C13	120.5 (2)
C10—C9—H9A	118.8	C17—C18—H18A	119.7
C8—C9—H9A	118.8	C13—C18—H18A	119.7
			
C10—C1—C2—C3	179.1 (3)	C6—C1—C10—C11	−177.6 (2)
C6—C1—C2—C3	−0.9 (4)	C9—C10—C11—C12	−104.1 (3)
C1—C2—C3—C4	1.4 (4)	C1—C10—C11—C12	74.6 (3)
C2—C3—C4—C5	−1.5 (5)	C13—N1—C12—O1	5.2 (4)
C3—C4—C5—C6	1.1 (5)	C13—N1—C12—C11	−172.3 (2)
C4—C5—C6—C7	−180.0 (3)	C10—C11—C12—O1	23.2 (3)
C4—C5—C6—C1	−0.6 (4)	C10—C11—C12—N1	−159.3 (2)
C10—C1—C6—C7	−0.1 (4)	C12—N1—C13—C18	−50.8 (3)
C2—C1—C6—C7	179.9 (2)	C12—N1—C13—C14	128.9 (3)
C10—C1—C6—C5	−179.5 (3)	C18—C13—C14—C15	−0.6 (3)
C2—C1—C6—C5	0.4 (4)	N1—C13—C14—C15	179.7 (2)
C5—C6—C7—C8	178.9 (3)	C18—C13—C14—Br1	178.87 (17)
C1—C6—C7—C8	−0.5 (4)	N1—C13—C14—Br1	−0.8 (3)
C6—C7—C8—C9	0.0 (4)	C13—C14—C15—C16	1.7 (3)
C7—C8—C9—C10	1.1 (4)	Br1—C14—C15—C16	−177.77 (17)
C8—C9—C10—C1	−1.7 (4)	C14—C15—C16—C17	−1.5 (3)
C8—C9—C10—C11	177.0 (2)	C15—C16—C17—C18	0.2 (4)
C2—C1—C10—C9	−178.8 (2)	C16—C17—C18—C13	0.9 (4)
C6—C1—C10—C9	1.2 (4)	C14—C13—C18—C17	−0.7 (3)
C2—C1—C10—C11	2.5 (4)	N1—C13—C18—C17	179.0 (2)

### Hydrogen-bond geometry (Å, º)

**Table:**

*D*—H···*A*	*D*—H	H···*A*	*D*···*A*	*D*—H···*A*
N1—H1*N*1···O1^i^	0.86 (3)	2.03 (3)	2.855 (3)	163 (2)
C11—H11*A*···O1^i^	0.99	2.55	3.279 (3)	130

Symmetry code: (i) *x*−1, *y*, *z*.

## References

[bb1] Bernstein, J., Davis, R. E., Shimoni, L. & Chang, N.-L. (1995). *Angew. Chem. Int. Ed. Engl.* **34**, 1555–1573.

[bb2] Bruker (2009). *APEX2*, *SAINT* and *SADABS* Bruker AXS Inc., Madison, Wisconsin, USA.

[bb3] Cosier, J. & Glazer, A. M. (1986). *J. Appl. Cryst.* **19**, 105–107.

[bb4] Flack, H. D. (1983). *Acta Cryst.* A**39**, 876–881.

[bb5] Fun, H.-K., Quah, C. K., Narayana, B., Nayak, P. S. & Sarojini, B. K. (2011*a*). *Acta Cryst.* E**67**, o2926–o2927. 10.1107/S1600536811041110 PMC3247340 22219958

[bb6] Fun, H.-K., Quah, C. K., Narayana, B., Nayak, P. S. & Sarojini, B. K. (2011*b*). *Acta Cryst.* E**67**, o2941–o2942. 10.1107/S1600536811041468 PMC3247353 22219971

[bb7] Fun, H.-K., Quah, C. K., Nayak, P. S., Narayana, B. & Sarojini, B. K. (2012). *Acta Cryst.* E**68**, o1385. 10.1107/S1600536812014869 PMC3344513 22590275

[bb8] Fun, H.-K., Quah, C. K., Vijesh, A. M., Malladi, S. & Isloor, A. M. (2010). *Acta Cryst.* E**66**, o29–o30. 10.1107/S1600536809051368 PMC2980099 21580136

[bb9] Sheldrick, G. M. (2008). *Acta Cryst.* A**64**, 112–122. 10.1107/S0108767307043930 18156677

[bb10] Spek, A. L. (2009). *Acta Cryst.* D**65**, 148–155. 10.1107/S090744490804362X PMC2631630 19171970

